# Toward Energy-Efficient Routing of Multiple AGVs with Multi-Agent Reinforcement Learning

**DOI:** 10.3390/s23125615

**Published:** 2023-06-15

**Authors:** Xianfeng Ye, Zhiyun Deng, Yanjun Shi, Weiming Shen

**Affiliations:** 1 School of Mechanical Science and Engineering, Huazhong University of Science and Technology, Wuhan 430074, China; xfye@hust.edu.cn (X.Y.); dengzy@hust.edu.cn (Z.D.); 2 Department of Mechanical Engineering, Dalian University of Technology, Dalian 116023, China; syj@dlut.edu.cn

**Keywords:** automated guided vehicles, multi-agent reinforcement learning, task assignment, path planning, energy consumption

## Abstract

This paper presents a multi-agent reinforcement learning (MARL) algorithm to address the scheduling and routing problems of multiple automated guided vehicles (AGVs), with the goal of minimizing overall energy consumption. The proposed algorithm is developed based on the multi-agent deep deterministic policy gradient (MADDPG) algorithm, with modifications made to the action and state space to fit the setting of AGV activities. While previous studies overlooked the energy efficiency of AGVs, this paper develops a well-designed reward function that helps to optimize the overall energy consumption required to fulfill all tasks. Moreover, we incorporate the e-greedy exploration strategy into the proposed algorithm to balance exploration and exploitation during training, which helps it converge faster and achieve better performance. The proposed MARL algorithm is equipped with carefully selected parameters that aid in avoiding obstacles, speeding up path planning, and achieving minimal energy consumption. To demonstrate the effectiveness of the proposed algorithm, three types of numerical experiments including the ϵ-greedy MADDPG, MADDPG, and Q-Learning methods were conducted. The results show that the proposed algorithm can effectively solve the multi-AGV task assignment and path planning problems, and the energy consumption results show that the planned routes can effectively improve energy efficiency.

## 1. Introduction

Automated guided vehicles (AGVs) are autonomous portable robots that navigate predetermined paths using various sensing technologies [1,2]. They play a vital role in modern manufacturing and logistics systems by facilitating the transportation of raw materials and finished products [3,4]. To optimize their performance, it is necessary to integrate the scheduling control system of AGVs with existing production management systems, such as manufacturing execution systems (MES), enterprise resource planning (ERP), warehouse management systems (WMS), logistics control systems (LCS), and Radio-Frequency IDentification (RFID) [5,6,7,8]. The AGV scheduling system receives request messages from the MES, dispatches AGVs to transport raw materials or finished products, and designs routes for AGVs to follow. However, the task assignment and path planning problem for multiple AGVs is challenging, as the number of decision variables and safety-related constraints grows significantly with the number of AGVs [9].

To address this issue, several optimization-based approaches have been proposed, such as integer programming [10,11,12], heuristic algorithms [13,14,15], and metaheuristics [7,16,17,18,19]. However, these methods have limitations in dealing with the dynamic and uncertain nature of the industrial environment. Therefore, machine learning-based methods, particularly reinforcement learning (RL), have emerged as a promising approach to solving the problem [20,21]. RL is a subfield of machine learning that involves an agent learning from its interactions with the environment to maximize a cumulative reward signal. Multi-agent reinforcement learning (MARL) is an extension of RL that involves multiple agents learning to coordinate with each other to achieve a common objective [22]. MARL has been shown to be effective in solving complex problems that involve coordination and competition among multiple agents [8].

Compared with existing studies, the key contributions of this paper are summarized as follows: (1) We propose an ϵ-greedy MADDPG algorithm which is able to converge faster and achieve better performance during training by balancing exploration and exploitation. (2) Modifications are made to the action and state space to fit the setting of AGV activities, while a well-designed reward function is incorporated into the proposed algorithm that optimizes energy consumption while fulfilling all tasks. (3) The effectiveness of the proposed algorithm is demonstrated through numerical experiments, which show that it outperforms other methods in improving energy efficiency while addressing the multi-AGV task assignment and path planning problem.

The remainder of this paper is organized as follows. Section 2 presents a literature review about AGV scheduling algorithms with MARL. Section 3 presents the background of reinforcement learning, followed by a description of the proposed algorithm and model in Section 4. Section 5 presents the simulation results and analyses. Section 6 concludes this paper and discusses some open issues and future work.

## 2. Literature Review

Reinforcement learning (RL) has become a promising solution to the AGV scheduling and routing problems, while many researchers have carried out much pioneering work with the application of RL [23].

For example, the Markov decision process (MDP) formulation was combined with the asynchronous deep Q network (DQN) to solve the routing problem in real time and obtain high-quality solutions [24]. A decentralized framework for multiple AGVs was proposed in [25] for multi-task allocation with attention (MTAA), which uses the DNN network and the A3C and MTAA-DQN path planning techniques to achieve task assignment equilibrium. Aside from this application, RL was used to solve the routing problem in a bidirectional transport network for the purpose of avoiding deadlocks and obtaining collision-free trajectories [26]. The deep Q network (DQN) was used in [27] to learn a transportation strategy with breakpoint continuation and hierarchical feedback, which can calculate and further modify a transportation schedule in a short time to accommodate dynamic factors. That aside, the authors of [28] tried to teach a neural network to allocate transportation duties to AGVs and design routes for them in accordance with the rewards computed by the network. An enhanced DQN was suggested in [29] to find appropriate navigational approaches for certain current road circumstances, which limits the Q output of specific actions and incorporates their outcomes using calculations based on experience-based pooling.

Moreover, a state space filter was proposed in [30] to improve the negotiation rules between different agents that adjust their routes when probable collisions are identified. Li et al. [31] proposed a deep learning approach that concurrently addresses task assignment and path planning concerns, and it uses the Markov decision chain to formulate the challenge of finding the shortest path without running afoul of other AGVs. In addition, De Ryck et al. [1] gave a general overview of the control algorithms and methods applied to the first-generation and latest AGV systems. Xue et al. [32] used an RL approach to solve a multi-AGV flow-shop scheduling problem, where AGVs communicate comprehensive data about each machine’s current state and running jobs. In other words, users are able to make decisions based on knowledge of the entire flow shop. Nagayoshi et al. [33] presented a decentralized autonomous strategy for controlling a large number of AGVs in response to ambiguous delivery requests, where the AGVs are equipped with transportation route plans that are intended to save travel time while avoiding collisions. Sierra-Garcia et al. [34] presented an intelligent hybrid control scheme that combines RL-based control (RLC) with conventional PI regulators, where the RLC allows the AGVs to learn how to improve trajectory tracking adaptively.

As suggested in [35], the multi-agent reinforcement learning (MARL) policy is capable of (1) scaling to a large number of agents in a real-world setting with an offline response time within acceptable levels and (2) outperforming existing algorithms with lower path lengths and faster solution times. Takahashi et al. [36] provided a multi-agent deep deterministic policy gradient (MADDPG) approach for managing several AGVs using DRL, where simulated experiments demonstrated that the suggested method learns optimal or nearly optimal solutions from prior knowledge. Aside from that, several numerical tests were carried out in [8] to confirm the effectiveness of the RL method. The authors of [37] used the MARL method to deal with the increased flexibility and complexity introduced by the increased use of AGVs. In addition, Li et al. [38] proposed a reward-shaping technique based on the potential information field which offers stepwise incentives and implicitly directs the AGVs to various targets to address the problem of reward sparsity. Moreover, Lu et al. [24] presented a DRL technique to address the AGV routing issue, where the conflict vectors are created from the retrieved embeddings and then processed using the LSTM network.

From the above work, it can be seen that RL is very effective for solving the AGV scheduling and routing problem. However, the existing DQN algorithm in RL has some limitations, since it cannot solve continued questions directly. Moreover, it does not consider how to solve the path planning problem with the application of RL.

## 3. Background

### 3.1. Single-Agent Reinforcement Learning Model

**Figure 1:**
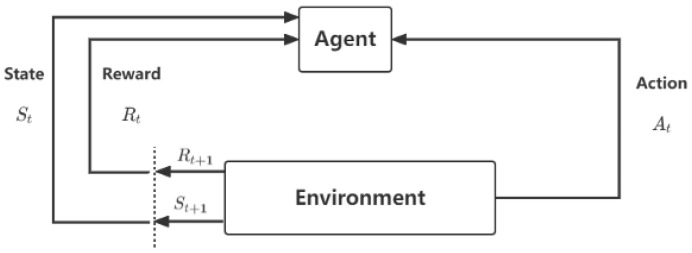
The single agent of the reinforcement learning system.

RL is a framework for learning how an agent can take action in an environment to maximize a cumulative reward signal. This framework can be expressed as a system consisting of an agent and an environment, as illustrated in Figure 1 [39]. The environment produces information that describes the state of the system, while the agent interacts with the environment by observing the state and then selecting an action to perform. Subsequently, the environment accepts the action and transitions into the next state while returning a reward to the agent. This reward denotes the feedback signal from the environment, indicating whether it is beneficial for the agent to adopt a certain strategy at a certain step. The agent’s objective is to learn a policy that maps states to actions to maximize the expected future cumulative reward. That is to say, the agent outputs the action $A_{t}$, observes the system’s state $S_{t}$, and receives the reward $R_{t}$ from the system.

In the context of AGVs, the vehicle can be formulated as an agent, since it can capture information with onboard sensors and perform an action with its actuators. The environment can be the map of a manufacturing factory or an automated warehouse where the AGV operates. The action space of the AGV agent can be represented by go forward, turn left, turn right, and stopping operations, for example. During the training phase, the agent interacts with the environment until the terminal conditions are met. After that, the agent can learn to perform actions without human guidance to maximize its expected future reward in a certain state.

### 3.2. Multi-Agent Reinforcement Learning Model

**Figure 2:**
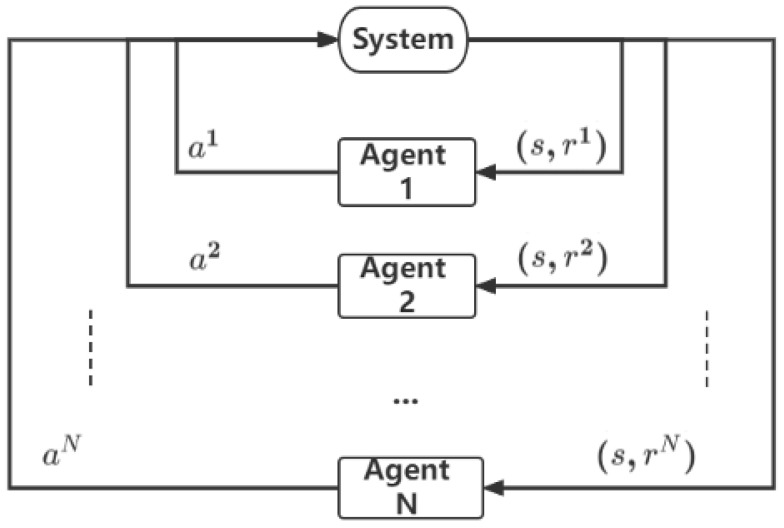
The multi-agent architecture in the reinforcement learning system.

In the MARL model, there are at least two agents existing in the same environment and interacting with each other as shown in Figure 2 [39]. We take into account the multi-agent Markov decision process extension known as Markov games [40]. A set of states *S*, action sets for each of *N* agents $A_{1},\ldots ,A_{N}$, a state transition function $T:S\times A_{1}\times \ldots A_{N}->P\left(S\right)$ which specifies the probability distribution over the possible next states, given the current state and actions for each agent, and a reward function for each agent that also depends on the overall state and actions of all agents $R_{i}:S\times A_{1}\ldots A_{N}->R$ define them. This means that all agents choose actions $a_{i}$ simultaneously after watching the system’s state s and receiving each agent’s individual reward $r_{i}$. In the case of multiple AGVs in a warehouse, each AGV can be considered an agent, and the entire warehouse map can be viewed as the environment.

To enable effective cooperation and competition between AGVs, researchers have developed various MARL algorithms that can learn the best strategies for multiple agents in the same environment. For example, one approach is to use a centralized training and decentralized execution (CTDE) architecture in which a central controller learns a joint policy for all agents during training, and each agent executes its own policy during execution. This approach has been shown to be effective in scenarios where there is a strong interdependence between agents, such as in a convoy of AGVs transporting a large item. Another approach is to use independent reinforcement learning (IRL), in which each agent learns its own policy independently without any communication or coordination with other agents. This approach can be useful when the actions of different agents do not have a significant impact on each other, such as in scenarios where AGVs are used to transport different items to different locations. MARL has the potential to improve the efficiency and effectiveness of AGV systems in various industrial applications, and ongoing research in this area is expected to lead to even more sophisticated and effective algorithms in the future.

## 4. Methodology

### 4.1. The Multi-Agent Deep Deterministic Policy Gradient (MADDPG) Algorithm

In this paper, we propose an energy-efficient scheduling and routing algorithm based on the multi-agent deep deterministic policy gradient (MADDPG) algorithm and the path planning D* Lite algorithm. The D* Lite algorithm’s basic idea is to plan the global optimal path from the destination point to the beginning point based on available environmental information, treating the unknown portion as free space [41]. However, in this paper, we combine the D* Lite algorithm with energy consumption computation to optimize energy efficiency.

In RL, the deep deterministic policy gradient (DDPG) algorithm is a model-free, off-policy, and policy-based method suitable for solving such problems [42,43]. The DDPG algorithm uses a deterministic policy, which means that when the policy and observed state are given, the action is uniquely determined. This is in contrast to classical RL algorithms, which use a stochastic policy that performs actions based on a probability distribution. DDPG follows the idea of fixing the target network that is used in the DQN algorithm, resulting in only two networks that need to be learned: the policy network and the value network [44,45]. In DDPG, each network is subdivided into a current network and a target network, and the updating process for these two networks is different. Under the actor-critic framework, the policy network is referred to as an actor network that outputs a deterministic action, while the value network is referred to as a critic network that fits the value function $Q_{\pi }\left(s,a\right)$. Multi-agent in DDPG, an extension of DDPG, means that decentralized agents learn a centralized critique based on their collective observations and actions, resulting in a multi-agent policy gradient algorithm. It generates learned policies that, during execution, only use local information (i.e., their own observations), does not require a differentiable model of the dynamics of the environment or any particular structure on the method of communication between agents, and is applicable to competitive or mixed interactions involving both physical and communicative behavior. The critic possesses additional knowledge about the practices of other agents, but the actor just has access to local information. Once trained, only locally based actors who work independently are used throughout the execution phase.

The estimated policy network of the actor is θ(s), where θ is the parameter of the neural network. The actor also has another target network that is used to update the value of the critic network. Both networks have the same structure and output corresponding actions, but the parameters within the neural networks are different. In terms of the critic network, there are also two networks: an estimation network and a target network. Both networks output the *Q* value of the current state, but they differ in terms of their input. For instance, the input of the critic’s target network has two parameters, which are the observation of the current state and the action of the actor’s target network output. In contrast, the input of the critic’s estimation network is the action of the current actor’s estimation network output.

The target network is used to calculate $Q_{target}$. The update of the value network is based on the gradient descent of the TD-error. The critic, which acts as a judge, does not initially know whether the actor’s action is good enough and needs to learn step by step to provide accurate scoring. With the help of the value $Q_{\pi }$ in the next moment, fitted by the target network and the actual gain *r*, we can obtain $Q_{target}$, which is then subtracted from the current *Q* to find the mean squared deviation, allowing us to construct the loss function. In terms of the policy network, its update is based on gradient ascent. Since the goal of the actor is to find an action *A* that maximizes the value *Q* of the output, optimizing the gradient of the policy network is for maximizing this *Q* value of the output of the value network. The loss function then adds a negative sign to facilitate minimizing the error.

The parameters of *n* agents are identified as $\theta =[\theta_{1},\ldots ,\theta_{n}]$, and the policies of *n* agents are identified as $\pi =[\pi_{1},\ldots ,\pi_{n}]$ [42]. Therefore, the accumulated reward for a certain agent *i* and the expected reward gradient for a deterministic policy $\mu_{\theta_{i}}$ can be represented as follows:

(1) $$J(\theta_{i})=E_{s∼\rho^{\pi },a∼\pi_{\theta_{i}}}\left[\sum_{t=0}^{\infty }\gamma^{t}r_{i,t}\right]$$

(2) $$L\left(\theta_{i}\right)=\frac{1}{s}\sum_{j}{\left(y^{j}-Q_{i}^{\mu }\left(x^{j},a_{1}^{j},\ldots ,a_{N}^{j}\right)\right)}^{2}$$

(3) $$\nabla_{\theta_{i}}J(\mu_{i})=E_{x,a∼D}\left[\nabla_{\theta_{i}}\mu_{i}\left(a_{i}|o_{i}\right)\nabla a_{i}Q_{i}^{\mu }\left(x,a_{1},\ldots ,a_{n}\right)|a_{i}=\mu_{i}\left(o_{i}\right)\right]$$

where $o_{i}$ is the observation of the agent *i*, $x=[o_{i},\ldots ,o_{n}]$ is the observation value, $Q_{i}^{\mu }\left(x,a_{1},\ldots ,a_{n}\right)$ is the action and state function, $\nabla_{\theta_{i}}\mu_{i}\left(a_{i}|o_{i}\right)$ is the gradient of the policy network at $\theta_{i}$, and $\nabla_{a_{i}}Q_{i}^{\mu }\left(x,a_{1},\ldots ,a_{n}\right)$ is the gradient of the value network at *x* and action sets $\left(a_{1},\ldots ,a_{n}\right)$.

### 4.2. Multi-Agent Model for AGV Operations

This paper presents a formal definition of the action space of the automated guided vehicle (AGV) agent, denoted by a(v,ω). The velocity of the AGV *v* is variable and can range from −1 m/s to 1 m/s, while the angular velocity ω is restricted to values between −1 rad/s and 1 rad/s. Consequently, the AGV agent is capable of performing five distinct actions, namely moving forward, moving backward, turning left, turning right, and halting. The reward function is defined as follows:

(4) $$r_{i}=k_{i1}\times D_{position}+k_{i2}\times C_{v}\times v\times cos\left(\omega \right)+k_{i3}\times C_{e}\times \left(E_{target}-E_{i}\right)+k_{i4}\times C_{AGV}+k_{i5}\times C_{obstacles}$$

In the above equation, $k_{i1}$, $k_{i2}$, $k_{i3}$, $k_{i4}$, and $k_{i5}$ are the weight parameters, while $D_{position}$ represents the reward value based on the current position relative to the previous position. A positive reward is given if the current position is closer to the destination than the previous position, and vice versa. This incentivizes the AGV to approach the target site, using the distance reward as guidance. The equation $D=\sqrt{{\left(x_{2}-x_{1}\right)}^{2}+{\left(y_{2}-y_{1}\right)}^{2}}$ calculates the distance between the current position and the destination point. The value of the award will be negative if $D_{current}$ is greater than $D_{previous}$. The value of the award will be negative if there is an AGV collision. Let us define the previous and current position for $AGV_{i}$ as $Pos1(x_{0},y_{0})$ and $Pos2(x_{1},y_{1})$ for $AGV_{j}$, respectively; that is, $Pos1(x_{2},y_{2})$ and $Pos2(x_{3},y_{3})$ if $\sqrt{({\left(x_{1}-x_{3}\right)}^{2}+{\left(y_{1}-y_{3}\right)}^{2})}<0.5$ are satisfied by $\left(y_{1}-y_{0}\right)\times \left(y_{3}-y_{2}\right)<0$ or $\left(x_{1}-x_{0}\right)\times \left(x_{3}-x_{2}\right)<0$, which means that if they travel in the opposite direction, then they will collide, and the reward value will be negative. The result of $\sqrt{\left({\left(x_{i}-x_{j}\right)}^{2}+{\left(y_{i}-y_{j}\right)}^{2}\right)}$ is less than 0.1 if an AGV collides with an obstruction due to the line’s narrow width. $C_{AGV}$ and $C_{obstacles}$ are both defined as −50 when there is a collision; otherwise, they are 50.

The speed reward guides the AGV to complete the task with the least number of rotations and the most significant amount of linear speed achievable. $C_{v}$ is the velocity coefficient and is used to scale this reward item. The third reward item is related to energy consumption, denoted by $E_{i}$, which is computed differently based on whether the AGV is stationary or in motion. When the AGV is not stationary, the energy consumption is computed as the average energy consumption per time step. Otherwise, the corresponding energy consumption is multiplied by a parameter factor of 0.3, which signifies that the AGV consumes less energy when in a stationary state compared with when it is in a normal driving state. The value of $E_{target}$ is computed using the route path determined by the D* Lite algorithm, while $C_{e}$ is the energy coefficient, which is used to fit this reward item with other items.

The last two reward items are related to collision avoidance and incentivize the AGV to avoid path conflicts with other AGVs or obstacles. These reward items are critical to ensuring the safe and efficient operation of the AGV. In addition, it is worth noting that the parameters in the reward function play a crucial role in numerical simulation and will be discussed further in another paper.

### 4.3. MADDPG with ϵ-Greedy

Exploration and exploitation are very prominent problems in reinforcement learning, and they are also the focus of determining whether the reinforcement learning system can obtain an optimal solution. Exploration would allow an agent to enhance its knowledge about its action, which may lead to long-term benefits. Improving the accuracy of action value estimation enables an agent to make more informed decisions. Exploitation uses the greedy action to acquire the greatest reward through exploiting the agent’s action value estimates. However, being greedy may not lead to the greatest reward and in fact may lead to suboptimal results. While exploration may find more accurate action value estimates, exploitation may obtain more rewards. However, it is not possible to do both at the same time. Exploration is the right way to maximize the expected return at the present moment, while exploitation is the right way to maximize the total return in the long run. Unfortunately, in a certain state, the agent can only perform one action: either exploration or exploitation. The two cannot be carried out at the same time, and thus this is the contradiction that accentuates the emphasis of reinforcement learning and how to balance exploration and exploitation.

The ϵ-greedy policy is a popular strategy for balancing exploration and exploitation [46,47]. This policy selects the best action with a probability 1−ϵ and a random action with a probability ϵ. The parameter ϵ determines the degree of exploration versus exploitation, where a high value of ϵ results in more exploration and a low value of ϵ results in more exploitation [48]. However, the ϵ-greedy method has a limitation in that it selects random actions uniformly, even though certain actions may be better than others. To address this limitation, softmax policies have been proposed, which select random actions with probabilities proportional to their current values [49]. In the context of AGV route selection, the ϵ-greedy policy can be used to balance exploration and exploitation by randomly selecting between exploration and exploitation. When one AGV explores its action, the other AGVs can exploit that action to their advantage. However, the optimal action for one AGV may not be the optimal action for all AGVs, as route conflicts may require different actions. Therefore, the challenge in AGV route selection is to find a balance between exploration and exploitation that maximizes the overall performance of the system. The ϵ-greedy policy is a straightforward strategy for balancing discovery and exploitation, where the parameter ϵ controls the degree of exploration versus exploitation. The algorithm of MADDPG with ϵ-greedy for AGVs is illustrated in Algorithm 1.
**Algorithm 1:** An algorithm of MADDPG with the ϵ-greedy policy for AGVs.   **for**
j=1 to max-episode **do**      Initialization of the parameters      **for**
t=1 to M **do**      **for**
i=1 to N **do**       n = random number       **if**
n<ϵ
**then**         execute any action(a)       **else**         execute the action which maximizes $Q_{t}(a)$
*with*
1−ϵ        **end if**        $a_{i}=\mu_{\theta i}(o_{i})+N_{t}$        $a=(a_{i},\ldots ,a_{N})$        $r_{i}=k_{i1}\times D_{position}+k_{i2}\times C_{v}\times v\times cos\left(\omega \right)+k_{i3}\times C_{e}\times \left(E_{target}-E_{i}\right)+k_{i4}\times C_{AGV}+k_{i5}\times C_{obstacles}$      **end for**      **for** agent i=1 to N **do**        $y^{j}=r_{i}^{j}+\gamma Q^{\mu }{}_{i}^{\prime }(x_{j}^{\prime },a_{1}^{\prime },\ldots a_{N}^{\prime })$        $L\left(\theta_{i}\right)=\frac{1}{s}\sum_{j}{\left(y^{j}-Q_{i}^{\mu }\left(x^{j},a_{1}^{j},\ldots ,a_{N}^{j}\right)\right)}^{2}$        $\nabla_{\theta_{i}}J≃\frac{1}{s}\sum_{j}\theta_{i}\mu_{i}(o_{i}^{j})a_{i}Q_{i}^{\mu }(x^{j},a_{1}^{j},\ldots ,a_{i},\ldots ,a_{N}^{j})|a_{i}=\mu_{i}(o_{i}^{j})$      **end for**      **for**
i=1 to N **do**        $\theta_{i}^{\prime }<-\tau \theta_{i}+(1-\tau )\theta_{i}^{\prime }$      **end for**      **end for**   **end for**

### 4.4. Three Algorithms in This Experiment

In this study, we focus on evaluating the performance of three popular reinforcement learning algorithms in a specific scenario. The algorithms we considered were Q-learning, MADDPG, and enhanced MADDPG with the epsilon-greedy policy, which are described below.

First, Q-learning is a widely used algorithm that employs off-policy reinforcement learning to maximize rewards. The algorithm updates a Q table that stores the expected reward of each state-action pair. Although Q-learning is a model-independent technique, it can be prone to taking risks in real-world applications. Our study aims to investigate the strengths and limitations of Q-learning in the given scenario.

MADDPG, on the other hand, is a centralized, critic-based actor-critic approach that allows one to consider various reward functions. The algorithm has a critic for each agent, which increases the amount of data used in the learning process. We evaluated the performance of MADDPG and compared it with Q-learning to gain insights into the strengths and limitations of these two approaches.

Finally, we introduce enhanced MADDPG with the epsilon-greedy policy, which is a straightforward strategy for balancing exploration and exploitation. By randomly selecting between exploration and exploitation, the algorithm can achieve a balance between the two strategies. We compared the performance of enhanced MADDPG with the epsilon-greedy policy with that of Q-learning and MADDPG to evaluate the effectiveness of this approach in the given scenario.

Our study contributes to the existing literature on reinforcement learning by evaluating the performance of three popular algorithms in a specific scenario. The findings of this study can help researchers and practitioners select the most appropriate algorithm for a given problem and improve the overall performance of reinforcement learning algorithms.

## 5. Experiments and Results

### 5.1. Test Scenarios

**Figure 3:**
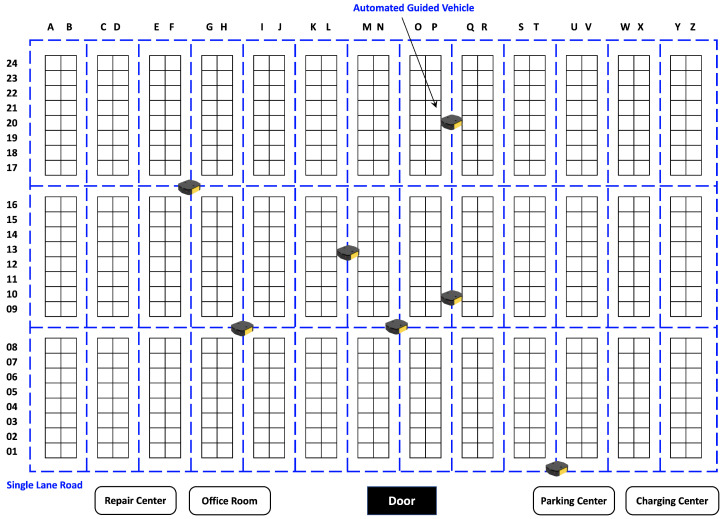
Map of the warehouse.

The experimental test scenarios presented in this paper aim to evaluate the performance of an AGV-based system in a warehouse environment. The state of the AGV includes the position, velocity, and distance between the starting point and the final destination. The warehouse, as illustrated in Figure 3, has a total area of 82×66 m^2^ and is equipped with a variety of facilities, including a power center, a repair center, and an office room for workers. The warehouse is divided into 39 racks, denoted by BLOCK, which are strictly off limits for AGVs.

The AGVs were programmed to stop at the park center when no tasks were assigned. Additionally, there were designated halt positions, such as A02 and B12, where the AGVs could temporarily pause while processing an order. There were three types of goods in this warehouse: delivered goods, transported goods, and relocated goods. Delivered goods were loaded at the door and unloaded at another position, whereas transported goods were moved from a specific rack to the door, and relocated goods were transferred from one rack to another. These tasks were challenging, as the AGVs had to navigate through the warehouse while avoiding obstacles, as shown in Figure 3.

To evaluate the effectiveness of the proposed algorithm, these tasks were selected as benchmark problems, and they are described in Table 1. The experimental scenarios aimed to examine the AGVs’ ability to perform these transfer tasks efficiently and accurately. The proposed algorithm’s performance was evaluated based on various metrics, including completion time, task efficiency, and AGV utilization. These experiments provided valuable insights into the AGV’s performance in a real-world warehouse environment, and they may lead to improvements in AGV-based systems’ efficiency and effectiveness.

### 5.2. Numerical Results

In this study, we analyzed the numerical results of the AGV system, which involved 30 AGVs and 3 types of goods categorized into 30 tasks, as illustrated in Table 1. Specifically, there were 12 tasks for delivered goods (Tasks 01–12), 12 tasks for transported goods (Tasks 13–24), and 6 tasks for relocated goods (Tasks 25–30). Each AGV was assigned 1 task, which required the 30 AGVs to complete all tasks simultaneously. For instance, AGV-01 was responsible for completing Task 01 by loading the cargo at the door and unloading it at position A24. The other tasks followed a similar pattern. Table 1 summarizes the three types of goods that needed to be processed. Two different types of software tools were used for the numerical simulations. A piece of the MADDPG algorithm was taken from [42] and specifically altered for our needs to yield the transportation line for the AGVs with parameters in Table 2. Our unique Java-written AGV-scheduling program was used in the left part for visualization of transportation.

**Figure 4:**
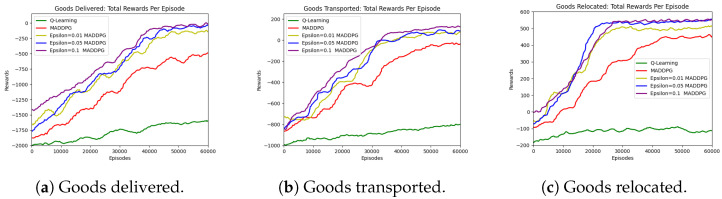
Three kinds of goods: rewards.

Figure 4 illustrates the convergence of the reward function in route selection based on the Q-learning technique. The function showed a gradual rise and eventual stabilization to a final state as the number of episodes increased. However, both the native multi-agent deep deterministic policy gradient (MADDPG) and ϵ-greedy MADDPG techniques converged more quickly to the steady state. When comparing the two MADDPG techniques, the ϵ-greedy strategy yielded better benefits. In the MADDPG algorithm, an ϵ value of 0.1 provided superior results to values of 0.01 and 0.05. This finding was consistent with most simulations of the ϵ-greedy approach, which demonstrated that an optimal value was attained around ϵ=0.1. However, determining the ideal value of ϵ for the current AGV environment requires further investigation.

**Figure 5:**
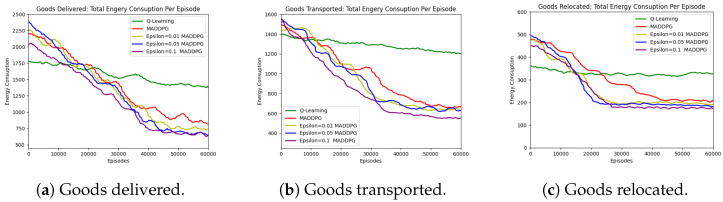
Three kinds of goods: energy consumption.

In this article, we used the AGV’s energy consumption per second as a unit, the AGV’s total energy once it arrived at its destination as an indicator, and the total energy of all AGV vehicles, as indicated in Figure 5. From this, it is clear that the route planned by Q-learning used a significant amount of energy, followed by the route planned by the primeval MADDPG algorithm and the route planned by the ϵ-greedy MADDPG algorithm, which used the least amount of energy.

**Figure 6:**
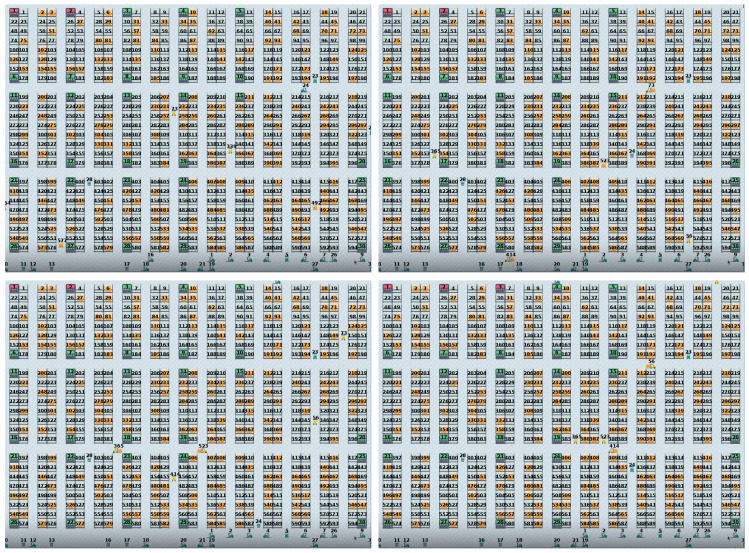
Illustration of AGVs’ routes.

The path of the AGV is shown in Figure 6. The shelves’ green tint indicates that they can hold stock. The shelf’s yellow tint shows that there is merchandise there. The shelves’ crimson tint indicates that they are completely full. When an AGV is in transit, it is shown by a yellow AGV, and when it is idle, it is indicated by a green AGV. From the figure, we can see that due to route conflicts, the AGV transporting items stopped for a period of time in the middle so that the transport AGV could pass smoothly. At the same time, some AGVs chose another path to avoid route conflicts.

Furthermore, the simulation results indicate that energy consumption should be taken into account when selecting the path for the AGVs. It was observed that the rewards fluctuated more when the AGVs traversed multiple obstacles, whereas the rewards were more stable when the AGVs encountered fewer obstacles.

## 6. Conclusions

In this paper, we proposed a multi-agent reinforcement learning (MARL) algorithm to address the problem of scheduling and routing multiple AGVs with the aim of minimizing the overall energy consumption. The proposed algorithm was built upon the multi-agent deep deterministic policy gradient (MADDPG) algorithm, with modifications made to the action space and state space to suit the specific activities of AGVs. While prior studies have overlooked the energy efficiency of AGVs, we designed a reward function that helps to optimize the overall energy consumption of the system during task completion.

To enhance the performance of our proposed MARL algorithm, we selected suitable parameters that facilitated obstacle avoidance, speedy path planning, and energy conservation. We conducted numerical experiments to evaluate the performance of the algorithm, and the results demonstrate its effectiveness in solving the multi-AGV task assigning and path planning problem. This leads to a reduction in the total energy consumption of AGV transportation, which increases as the number of operational AGVs increases.

During the simulation, the outcomes of the simulation were influenced by a range of parameters. We adopted identical settings for all AGVs in the reward function, although the parameters for each AGV’s reward function were slightly different due to variations in position and the actions taken in response to those positions. These differences arose due to the unique nature of each AGV’s activities. For instance, an AGV tasked with transporting heavier goods may use more energy than one carrying lighter items. Additionally, we observed that the optimal ϵ value for the MADDPG model in the current environment has not yet been established using the ϵ-greedy approach.

In our future work, we plan to develop an end-to-end learning framework that focuses on the direct control inputs of AGVs, rather than utilizing the desired velocity and angular velocity as the action space. We anticipate that this approach will enhance the performance of our proposed algorithm even further. By integrating a more comprehensive understanding of AGVs’ behaviors and their interactions with the environment, the algorithm will be better equipped to adapt to the diverse requirements of various tasks and settings. Ultimately, we are confident that our research will contribute significantly to the advancement of energy-efficient AGV systems, which are of growing importance in contemporary logistics and transportation applications.

## Figures and Tables

**Table 1:** Thirty transfer tasks in three projects.

Transfer Type	Task List	Load Cargo Position	Unload Cargo Position
Goods Delivered	Task 01	Door	A24
	Task 02	Door	A17
	Task 03	Door	A16
	Task 04	Door	A09
	Task 05	Door	A08
	Task 06	Door	A01
	Task 07	Door	E24
	Task 08	Door	E17
	Task 09	Door	E16
	Task 10	Door	E09
	Task 11	Door	E08
	Task 12	Door	E01
Goods Transported	Task 13	I24	Door
	Task 14	I17	Door
	Task 15	I16	Door
	Task 16	I09	Door
	Task 17	I08	Door
	Task 18	I01	Door
	Task 19	M24	Door
	Task 20	M17	Door
	Task 21	M16	Door
	Task 22	M09	Door
	Task 23	M08	Door
	Task 24	M01	Door
Goods Relocated	Task 25	Q24	Z01
	Task 26	Q17	Z08
	Task 27	Q16	Z09
	Task 28	S24	W01
	Task 29	S17	W08
	Task 30	S16	W09

**Table 2:** Parameter settings for simulations.

Description	Notation and Value
Weight Parameters	$k_{i1}=0.2,k_{i2}=0.1,k_{i3}=0.5,k_{i4}=0.1,k_{i5}=0.1$
Reward Value	$D_{position}$
Velocity Coefficient	$C_{v}$
Energy Consumption	$E_{i}$
Target Coefficient	$E_{target}$
Energy Coefficient	$C_{e}$
Collision Parameter between AGVs	$C_{AGV}$
Collision Parameter between AGV and Obstacle	$C_{Obstacle}$
Learning Rate	0.15
Discount Factor	0.99
α	0.01
β	0.01
γ	0.95
τ	0.01

## Data Availability

The data presented in this study are available on request from the corresponding author. The data are not publicly available due to privacy.
